# DAMPs, PAMPs, and Alarmins: From Mechanism to Therapy

**DOI:** 10.1002/mco2.70923

**Published:** 2026-08-22

**Authors:** Xuanxuan Yu, Yuqin Jin, Baochao Li, Jie Deng, Yiwen Zhou, Jinglun Zhang, Huang Li

**Affiliations:** ^1^ Nanjing Stomatological Hospital Affiliated Hospital of Medical School Institute of Stomatology Nanjing University Nanjing China; ^2^ Department of Stomatology the First People's Hospital of Changzhou the Third Affiliated Hospital of Soochow University Changzhou China

**Keywords:** adaptive immunity, damage‐associated molecular patterns (DAMPs), innate immunity, pattern recognition receptors (PRRs), pathogen‐associated molecular patterns (PAMPs)

## Abstract

Immune surveillance hinges on the precise regulation of molecular sentinels to maintain the equilibrium between physiological homeostasis and pathological distress. Central to this process is the sensing of exogenous pathogen‐associated molecular patterns (PAMPs) and endogenous damage‐associated molecular patterns (DAMPs), or alarmins. Traditionally, PAMPs and DAMPs were categorized as triggers for local protective host defense or maladaptive inflammatory cascades. Recent insights have unveiled an expanded functional framework for these signals, where they orchestrate systemic processes, notably including trained immunity and interorgan communication. Despite these advances, existing reviews lack a unified synthesis that integrates classical functions with novel concepts. This review narratively synthesizes molecular sources, sensing mechanisms, and regulatory networks that control PAMP and DAMP signaling, followed by their diverse functions in modulating both immune and nonimmune landscapes. Critically, we dissect the intricate interplay between PAMPs and DAMPs, a dimension overlooked in prior reviews. We further discuss their contributions to various human diseases, highlighting their context‐dependent functions in different conditions. Finally, we evaluate their emerging role in biomarkers and therapies. By integrating classical concepts with recent discoveries, this review provides a comprehensive framework for understanding how danger signals shape human health and disease, offering insights for biomarker development and immunomodulatory therapies.

## Graphical abstract

This review synthesizes the expanding biology of PAMPs and DAMPs, from their molecular sources and sensing mechanisms to regulatory networks that govern inflammation and homeostasis. Spanning from classical immune activation to trained immunity and interorgan communication, we highlight the interplay between PAMPs and DAMPs and their context‐dependent roles across human diseases. Further, we evaluate their emerging utility as biomarkers and immunomodulatory therapies, offering a unified framework for translating danger signal biology into clinical practice.

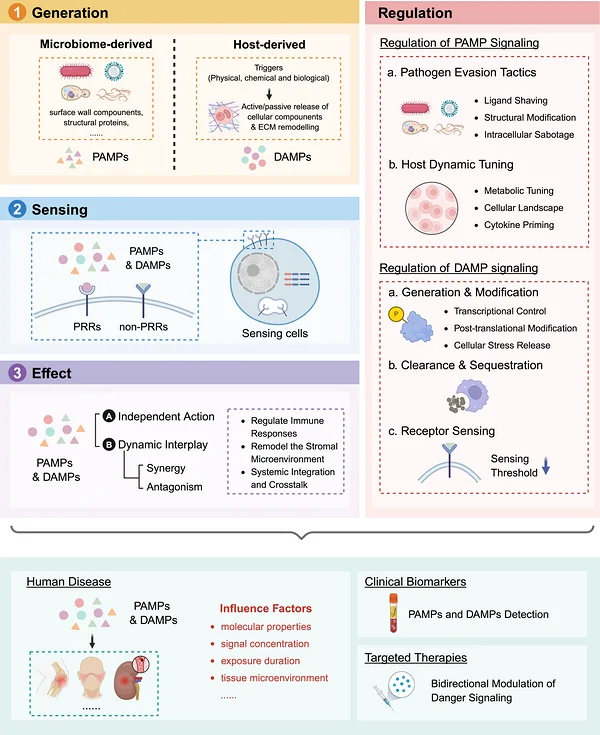

## 1 Introduction

The conceptual landscape of innate immunology has undergone a transformative evolution over the last three decades, shifting from a binary self/non‐self‐discrimination model to a multifaceted “danger” surveillance network. This paradigm shift began in 1989, when Charles Janeway proposed the concept of Pathogen‐Associated Molecular Patterns (PAMPs) [1]. PAMPs are defined as evolutionarily conserved, invariant molecular signatures, such as lipopolysaccharides (LPS) or double‐stranded RNA (dsRNA), that are unique to microorganisms and recognized by germline‐encoded pattern recognition receptors (PRRs).To address the limitations of the PAMP model in explaining sterile inflammation where PAMPs are absent, Polly Matzinger introduced the Danger Theory in 1994 [2], formalizing the role of damage‐associated molecular patterns (DAMPs), which were later defined by Water Land in 2003 [3]. Unlike PAMPs, DAMPs are endogenous molecules normally sequestered within healthy cells or tissues that are liberated into the extracellular milieu following cellular stress or cell death, thereby alerting the host to internal threats regardless of microbial presence. This nomenclature was further refined in 2005 by Joost Oppenheim, who introduced the term “alarmins” to describe a specific subset of endogenous mediators, such as HMGB1 and defensins, that are rapidly released upon tissue injury to recruit and activate immune cells [4]. Although distinct in their historical origins, the terms DAMPs and alarmins are utilized synonymously in the vast majority of current literature. Consequently, this review will incorporate alarmins into a unified narrative under the broader DAMP category to provide a more cohesive structural framework.

While PAMPs and DAMPs originate from distinct exogenous or endogenous sources, they are fundamentally interconnected, often converging on shared signaling pathways to coordinate the host's defensive and reparative programs. Crucially, these danger signals do not operate in isolation; rather, their functions are intricately intertwined, with PAMPs and DAMPs engaging in complex bidirectional crosstalk that shapes immune responses. Under physiological conditions, these signals facilitate the clearance of pathogens and debris and maintain homeostatic equilibrium [5]. However, their aberrant accumulation under pathological conditions drives the progression of diverse disorders, including chronic infections, autoimmune diseases, and malignancy [6]. In recent years, the scope of danger signal biology has expanded beyond these classical roles into revolutionary new frontiers. Recent evidence has positioned PAMPs and DAMPs as central architects of trained immunity [7, 8], a form of innate immune memory where primary exposure to these signals induces long‐term functional reprogramming through sustained metabolic and epigenetic modifications. Furthermore, these molecules are now recognized as essential mediators of cross‐organ crosstalk, functioning as systemic danger signals that traverse physiological barriers to communicate inflammatory status between disparate anatomical compartments, such as the gut–liver or brain–bone axes [9]. In addition, the therapeutic potential of targeting PAMP and DAMP pathways has garnered increasing attention [10]. Strategies range from inhibiting PAMP and DAMP signaling for controlling inflammatory response to harnessing their immunostimulatory properties for vaccination and cancer immunotherapy.

**FIGURE 1:**
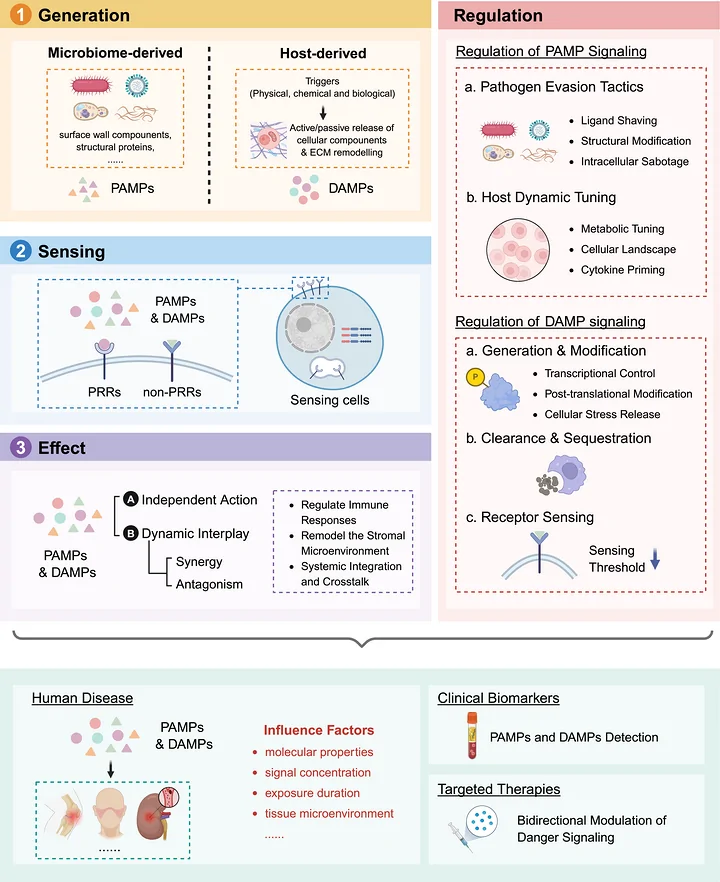
Integrative conceptual framework of PAMP and DAMP biology. The schematic illustrates the biological progression, regulatory networks, and clinical implications of danger signals. (1) Generation: Exogenous PAMPs originate from microbial components, while endogenous DAMPs are liberated via cellular stress or extracellular matrix (ECM) remodeling. (2) Sensing: These signals are detected by specialized sentinel cells equipped with pattern recognition receptors (PRRs) and non‐PRRs. (3) Effect: PAMPs and DAMPs function either independently or through dynamic interplay (synergy and antagonism) to regulate immune responses, remodel the stromal microenvironment, and drive systemic crosstalk. These signaling cascades are tightly calibrated: PAMP signaling is shaped by the tension between pathogen evasion tactics and host dynamic tuning, whereas DAMP signaling is strictly controlled at the levels of generation, clearance, and receptor sensing thresholds. The ultimate biological trajectory toward human disease is dictated by contextual influence factors. Translating these mechanistic insights facilitates the application of PAMPs and DAMPs as clinical biomarkers and guides the development of bidirectional targeted therapies for immune modulation.

To synthesize contemporary advancements, we critically evaluated English‐language literature concerning PAMPs and DAMPs published within the last five years across databases such as PubMed and EMBASE. Building upon this foundational literature, this review provides an in‐depth analysis of PAMP and DAMP molecular characterization, subcellular sensing, and regulatory networks. We further examine their multifaceted pathological roles and the clinical progress in therapeutic development, ultimately bridging the gap between basic biology of danger signals and translational applications (Figure 1).

## 2 Overview of PAMPs and DAMPs

To comprehend how the host immune system discriminates between physiological safety and pathological distress, it is essential to map the structural and functional organization of the danger surveillance network. This section provides a foundational overview of danger signal biology, delineating their structural attributes, detection mechanisms, and regulatory networks.

### 2.1 Molecular Characterization and Classification

The initiation of immune responses depends on the biochemical classification of the driving ligands. This subsection categorizes danger signals by their origin. Exogenous signals (PAMPs) consist of conserved structural motifs and metabolic intermediates unique to microorganisms. Endogenous signals (DAMPs and alarmins) are normally sequestered intracellular molecules that are released into the extracellular space through active secretion or various cell death pathways. Additionally, bioactive fragments derived from the extracellular matrix function as independent DAMPs. Collectively, these distinct molecules serve as primary alerts for localized or systemic homeostatic perturbations.

#### 2.1.1 PAMPs: The Exogenous Signatures

PAMPs represent highly conserved, evolutionarily stable molecular signatures generated by microorganisms that are intrinsically absent from the host proteome. Although the term “pathogen” implies a restriction to virulent microbes, these motifs are ubiquitously produced by both pathogenic and commensal species [10]. Consequently, the term Microbe‐Associated Molecular Patterns (MAMPs) is often considered a more semantically accurate descriptor [11]. For the sake of readability and consistency with the prevailing literature, however, we retain the original PAMP nomenclature throughout this review.

Several fundamental properties render PAMPs ideally suited for innate immune recognition [12]. First, they represent products of microbial‐specific pathways, providing unambiguous discrimination between self and non‐self for the host. Besides, they are conserved within entire classes of microorganisms, enabling a limited repertoire of germline‐encoded receptors to detect a vast array of potential pathogens. Crucially, they play indispensable roles in microbial physiology and survival, thereby imposing significant constraints on microorganisms' ability to evade innate immune recognition through adaptive evolution.

**FIGURE 2:**
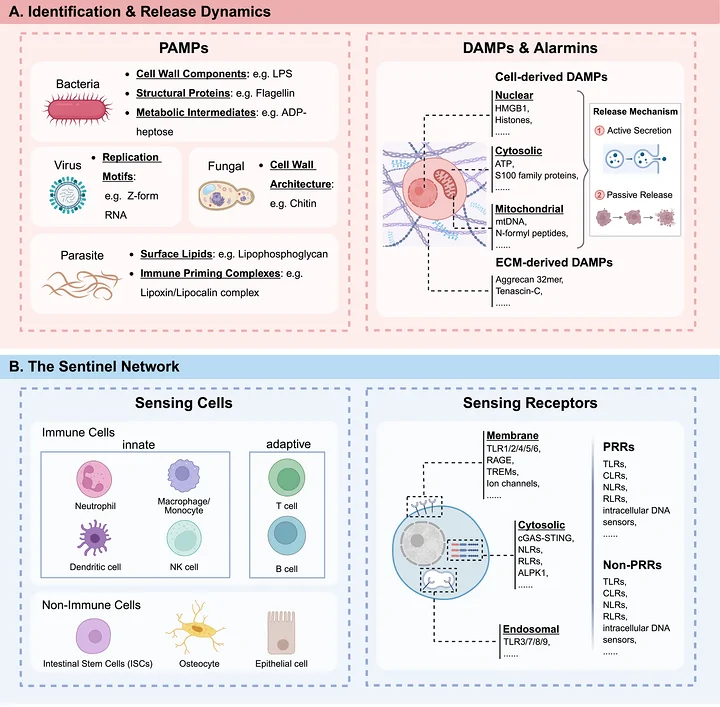
Molecular diversity, release mechanism, and the sentinel sensing network of PAMPs and DAMPs. (A) Identification and release dynamics. The PAMP/DAMP axis categorizes molecular danger signatures by their origin, exogenous microbial motifs (bacteria, viruses, fungi, and parasites) versus endogenous alarmins sequestered in intracellular or extracellular matrix (ECM) depots. Intercellular DAMPs are liberated through either active secretion or passive liberation. (B) The sentinel network: Danger perception is orchestrated by a hierarchical cellular network, comprising professional innate/adaptive leukocytes and nonimmune residents (e.g., ISCs, osteocytes), and a topographically organized receptor system. PRRs and non‐PRRs provide spatially partitioned surveillance across diverse subcellular locations.

PAMPs are produced by a broad spectrum of microorganisms, including bacteria, viruses, fungi, and parasites (Figure 2). Bacterial PAMPs predominantly comprise surface‐exposed components such as LPS from Gram‐negative species, lipoteichoic acids (LTA) from Gram‐positive strains, the structural protein flagellin, and bacterial nucleic acids [13]. Beyond these classical structural components, recent research has substantially expanded the PAMP landscape by identifying soluble metabolic intermediates as potent immune agonists. During lipopolysaccharide biosynthesis, Gram‐negative bacteria generate adenosine diphosphate (ADP)‐heptose, a highly conserved metabolic intermediate that functions as a previously unrecognized PAMP, entering the host cell cytosol to activate innate defenses through the cytosolic sensor ALPK1 [14]. Subsequent investigations have identified its derivatives, such as cytidine diphosphate (CDP)‐heptose and uridine diphosphate (UDP)‐heptose, as similarly potent innate immune agonists [15]. Given that functional heptose‐bisphosphate enzymes are distributed across bacteria, archaea, eukaryotes, and even certain viruses, β‐D‐manno‐heptose has been identified as a “cross‐kingdom” small molecule PAMP capable of triggering the ALPK1‐dependent inflammatory signaling cascade [15].

Because all viral components are synthesized within host cells using host machinery, viral nucleic acids constitute the principal targets for innate immune surveillance. Therefore, viral PAMPs consist primarily of unique nucleic acid motifs generated de novo during active replication cycles. For example, the influenza A virus, a lytic RNA virus, generates Z‐form RNA during its replication cycle; these Z‐RNA structures are recognized by the ZBP1 sensor within the nuclei of infected cells, triggering innate immune responses [16].

Fungal PAMPs are derived primarily from cell wall components. The fungal cell wall comprises two principal layers: the inner wall, composed of chitin and linear β‐glucans, and the outer wall, which consists of heavily glycosylated mannoproteins [17]. Under physiological conditions, this inner core is typically sequestered by the outer mannan layer to avoid immune recognition; however, its exposure during tissue injury or microbial turnover provides a critical signal for the initiation of the antifungal innate response [18]. The fungal cell wall exhibits remarkable compositional diversity, although certain structural elements, notably β‐glucans and chitin, are broadly conserved across fungal genera [18]. Therefore, these conserved components function as PAMPs, representing an inherent vulnerability in the fungal defensive architecture [19].

Beyond the well‐characterized molecular signatures of bacteria, viruses, and fungi, parasite‐derived PAMPs represent a structurally heterogeneous class of innate immune triggers [20]. These motifs are recognized across various developmental stages and exhibit significant biochemical diversity. For instance, during infection with Trypanosomatids such as *Trypanosoma cruzi* and *Leishmania*, the innate immune system utilizes Toll‐like receptor (TLR) 2 to detect surface‐associated alkylacylglycerol and lipophosphoglycan, while TLR9 serves as a specialized sensor for parasitic genomic DNA [21]. Furthermore, the host–parasite interface often involves the release of unique molecular assemblies that facilitate immune priming. A sophisticated example is the Lipoxin/Lipocalin complex liberated during *Plasmodium* midgut invasion [22]. While lipoxins are traditionally recognized for their pro‐resolving properties in vertebrates, this lipid–protein complex acts as a critical signal that heightens the responsiveness of the innate immune compartment, specifically through the activation and differentiation of phagocytic cells, to subsequent challenges.

#### 2.1.2 DAMPs and Alarmins: The Endogenous Danger Signals

DAMPs, namely alarmins, constitute the endogenous mediators of the danger model. Normally restricted to intracellular or extracellular depots, these molecules are unleashed into the surrounding milieu in response to pathological perturbation, thereby exerting their potent immunogenic potential [6]. Their generation is orchestrated by a triad of physical (e.g., excessive mechanical load or ionizing radiation), chemical (e.g., chemotherapy‐induced cytotoxicity), and biological (e.g., microbial invasion or immunosenescence) insults [6].

Intracellular DAMPs originate from multiple subcellular compartments (Figure 2). Nuclear DAMPs include HMGB1 and histones that acquire immunostimulatory properties upon extracellular release [23]. Cytosolic DAMPs encompass molecules like ATP and S100 family proteins [24]. Mitochondrial DAMPs, such as mitochondrial DNA, are particularly potent due to their evolutionary ancestry, containing unmethylated CpG motifs and N‐formylated methionine that function as molecular mimics of bacterial PAMPs [25, 26].

DAMPs exit cells through two fundamentally distinct kinetic routes: active secretion and passive release. Active release involves posttranslational modifications and vesicular trafficking, enabling controlled DAMP export without compromising membrane integrity. Cellular stress induces posttranslational modifications in DAMPs, including acetylation, methylation, phosphorylation, and lactylation, that enable their recognition and sequestration into vesicles [27]. Second‐messenger molecules, secretory autophagy, and metabolic reprogramming further regulate this active export pathway, enabling DAMPs to be packaged into extracellular vesicles that travel to distant organs without enzymatic degradation [28]. For example, lactate drives the acetylation and lactylation of HMGB1 by suppressing the deacetylase SIRT1 through Hippo/YAP signaling and facilitating the GPR81/β‐arrestin2‐mediated nuclear translocation of p300/CBP acetyltransferases, ultimately triggering the exosomal secretion of nuclear HMGB1 from macrophages to the extracellular region [29].

Passive release, conversely, occurs during lytic cell death when plasma membrane rupture allows uncontrolled efflux of intracellular constituents. Necrosis and necroptosis have long been recognized as sources of passive DAMP release [30]. Recent research found that in necroptosis, MLKL not only propagates inflammation through noncell‐autonomous DAMP release but also triggers cell‐autonomous inflammatory signaling by facilitating mitochondrial DNA leakage into the cytoplasm, thereby activating the cGAS‐STING pathway [31]. Beyond these well‐established modalities, recent research has revealed that other regulated cell death pathways contribute to distinct DAMP repertoires. During pyroptosis, inflammatory caspase activation cleaves gasdermin D, generating plasma membrane pores that enable selective efflux of DAMPs while preserving partial membrane integrity [32]. Ferroptosis, defined by iron‐dependent lipid peroxidation and subsequent membrane disruption, similarly creates a conduit for DAMP liberation, which is mediated by NINJ1 [33]. PANoptosis represents an integrated cell death pathway wherein simultaneous activation of multiple death programs, pyroptosis, apoptosis, and necroptosis, converges within a single cell under the coordination of the PANoptosome complex. This multifaceted death modality ensures robust DAMP release while eliminating cells that might otherwise harbor intracellular pathogens [34]. Beyond these immunogenic forms of cell death, emerging research has discovered that even apoptosis, historically considered immunologically silent due to preserved membrane integrity, can contribute to DAMP liberation. It has been found that apoptotic cells expose nuclear constituents on their surface and subsequently release these components into the extracellular environment [23].

In addition to intracellular sources, the extracellular matrix also constitutes an important source of DAMPs through structural rearrangements, such as tenascin‐C and aggrecan 32mer [35] (Figure 2). This process is driven by three primary mechanisms: alternative splicing of ECM components generating tissue‐specific isoforms [36], posttranslational modifications [37], and protease‐mediated degradation that liberates immunostimulatory fragments [38]. These ECM‐derived DAMPs further activate pattern recognition receptors, amplifying local inflammation.

### 2.2 Sensing Mechanism: The Cellular and Molecular Sentinel Network

The transition from physiological homeostasis to pathological inflammation is governed by a highly organized network of sensing platforms. This sensing system comprises diverse cell populations functioning as biological sentinels and an intricate topography of receptors that decode the biochemical language of danger (Figure 2).

#### 2.2.1 Sensing Cells

The detection of danger signals is a collective effort executed by a broad spectrum of cell types, ranging from professional innate sentinels to adaptive lymphocytes and nonimmune tissue residents. Innate immune cells, including macrophages [39], neutrophils [6], and dendritic cells (DCs) [40], constitute the primary defensive line. Besides, natural killer (NK) cells specifically employ receptors like NKp30 (NCR3) [41] and NKp46 [42] as “cytotoxic PRRs” to directly recognize fungal components or stress‐induced ligands. In addition to innate immune cells, adaptive immune cells such as T and B cells utilize TLRs (e.g., TLR2 and TLR5) to modulate their activation thresholds and functional output [43]. Furthermore, the concept of cell‐autonomous immunity has been broadened to include nonimmune populations, such as osteocytes [44], intestinal stem cells [45], and various epithelial cells [46, 47], which independently sense microbial translocation or tissue injury.

Recent advancements in single‐cell technologies have further refined our understanding by identifying highly specialized cell sub‐populations. For example, research identified the “first responder” dendritic cells within conventional DC populations, which are uniquely capable of reaching the activation threshold upon initial PAMP exposure and subsequently orchestrating the immune response through paracrine signaling to mobilize bystander cells [48]. The precision targeting of these “first responders” represents a transformative frontier in vaccine design, offering a means to maximize immunological efficiency while mitigating systemic adverse effects.

#### 2.2.2 Sensing Receptors

Danger perception is mediated by both PRR and non‐PRR sensors [49]. PRRs can bind to both PAMPs and DAMPs, which encompass TLRs and C‐type lectin receptors (CLRs), cytosolic NOD‐like receptors (NLRs), RIG‐I‐like receptors (RLRs), and multiple intracellular DNA sensors. Additionally, DAMPs can interact with non‐PRRs, which include the receptor for advanced glycation end products (RAGE), triggering receptors expressed on myeloid cells (TREMs), G protein‐coupled receptors (GPRs), and ion channels.

These receptors are organized according to distinct subcellular localizations. The TLR family exhibits distinct subcellular topography: TLR1, 2, 4, 5, and 6 are localized on the plasma membrane to monitor the extracellular space, whereas TLR3, 7, 8, and 9 are sequestered within endosomal membranes to survey the endocytic environment [50]. Cytosolic surveillance operates through sensors such as cGAS‐STING recognizing misplaced DNA [51] and ALPK1 detecting bacterial ADP‐heptose [45]. Mitochondria serve as signaling hubs, utilizing MAVS to integrate RNA sensing with bioenergetic adaptation [51]. To achieve optimal sensitivity and specificity, many sensors require specialized adaptor proteins or “two‐factor authentication” strategies to verify transient or low‐abundance signals. For instance, the extracellular co‐factor MD2 is essential for TLR4‐mediated sensing, illustrating that the fidelity of the sentinel network is fundamentally predicated on these intricate molecular docking and verification events [52]. Similarly, the detection of cytoplasmic HIV‐1 DNA, a low‐abundance PAMP, necessitates the adaptor protein PQBP1. Upon infection, PQBP1 decorates the viral capsid to provide primary verification, subsequently recruiting cGAS to the site of PAMP generation to initiate immune responses [53].

### 2.3 Regulation of PAMP and DAMP Signaling

The magnitude and duration of PAMP and DAMP signaling must be precisely calibrated to ensure effective host defense while preventing maladaptive immune responses. This regulatory imperative creates a dynamic tension: pathogens evolve strategies to minimize PAMP recognition and downstream signaling as a means of immune evasion, whereas the host must maintain robust sensing mechanisms to detect microbial invasion and endogenous danger signals while simultaneously avoiding excessive or sustained activation that could lead to tissue damage. The regulatory mechanism governing these pathways operates at multiple levels, from the generation and modification of the signals themselves to the expression and function of their cognate receptors.

#### 2.3.1 Regulation of PAMP Signaling

Pathogenic evolution has yielded an intricate repertoire of tactics designed to minimize the visibility of PAMPs, thereby subverting host surveillance. In *Candida albicans*, the highly immunostimulatory β‐1,3‐glucan is a potent PAMP that the host is primed to recognize [54]. However, upon sensing host‐derived metabolic cues such as lactate, the fungus initiates an active “shaving” mechanism by inducing the Xog1 exoglucanase [55]. This enzymatic removal of surface‐exposed glucans, co‐regulated by Protein Kinase A signaling, ensures efficient immune concealment and diminishes phagocytic recognition. Similarly, *Helicobacter pylori* achieves chronic persistence within the gastric mucosa through the structural modification of its LPS. By adding long‐chain fatty acyl groups to the Lipid A moiety, the pathogen renders its LPS largely invisible to TLR4, while simultaneously utilizing specialized flagellin sequences that bypass TLR5 detection [56]. Beyond ligand modification, certain pathogens exert direct sabotage on host intracellular cascades. For instance, specific bacterial toxins hijack host caspase‐3 to cleave the active N‐terminal gasdermin D, thereby suppressing macrophage pyroptosis and preserving a protected intracellular niche for replication [57]. Conversely, clinical interventions can be leveraged to forcibly restore PAMP visibility and enhance pathogen clearance. Tobramycin‐mediated bactericidal activity increases the systemic liberation of bioactive LPS, which in turn amplifies the localized inflammatory milieu and augments antibiotic efficacy through neutrophil‐dependent recruitment [58].

Reciprocally, the host maintains a dynamic sensing threshold by modulating pattern recognition receptors. Lipid mediators, such as epoxyeicosatrienoic acids, can attenuate the inflammatory response to *Streptococcus pneumoniae* by downregulating the expression of TLR2 and PGLYRP1, illustrating the host's ability to tune innate sensitivity via endogenous metabolites [59]. Furthermore, the overall cellular landscape profoundly dictates PAMP responsiveness. Senescent cells exhibit a hyperinflammatory primed phenotype. When challenged by PAMP motifs such as the SARS‐CoV‐2 spike protein, these senescent cells upregulate viral entry factors and suppress the antiviral defenses of neighboring nonsenescent cells through paracrine signaling cascades [60]. This contextual sensitivity is further calibrated by cytokine environments. For example, IFN‐γ priming utilizes caspase‐8‐dependent pathways to lower the physiological threshold for PAMP‐induced cell death, ensuring a rapid response to imminent threats [61].

#### 2.3.2 Regulation of DAMP Signaling

DAMP activity is regulated at three interconnected levels: generation, clearance, and sensing. These control mechanisms ensure that danger signals accurately reflect genuine tissue injury. However, a disruption in this balance results in persistent inflammation contributing to chronic pathology.

DAMP production is controlled through transcriptional, posttranslational, and cellular mechanisms. At the transcriptional level, DAMP expression is dynamically calibrated rather than constitutive; C/EBPδ drives epigenetic remodeling that governs *S100a8* and *S100a9* transcription in response to inflammatory signals [62]. Posttranslational modifications further modulate DAMP functionality: circulating FABP5 exists in an oxidized form that exacerbates septic inflammation as a DAMP, whereas its reduced cytoplasmic counterpart actively suppresses pyroptosis, demonstrating how oxidation state converts a benign intracellular protein into a pro‐inflammatory mediator [63]. Cellular stress pathways can influence DAMP liberation. Compound F1929‐1458 engages NF‐κB signaling in stressed tumor cells, triggering release of HMGB1‐genomic DNA complexes, ATP, and oxidized LDL (oxLDL) that activate dendritic cell cGAS‐STING and NLRP3 pathways [64].

To maintain immunological silence, the homeostatic concentration of extracellular DAMPs is regulated by clearance systems and active sequestration [65]. When the rate of cellular death overwhelms these efferocytic pathways, exemplified by liver injury where hepatocyte apoptosis saturates compromised macrophage efferocytic capacity, the resultant accumulation of secondary necrotic debris (originating from uncleared apoptotic cells) fuels a self‐perpetuating inflammatory cycle through the release of DAMPs like HMGB1, oxidized mtDNA, and bioactive phospholipids [66]. This clearance is orchestrated by molecular opsonins such as the apoptosis inhibitor of macrophages (AIM), which anchors to DAMPs via charge‐based and disulfide interactions to facilitate phagocytic uptake [67]. Beyond promoting debris removal, AIM provides a critical layer of immune regulation by sterically hindering DAMP‐receptor engagement, thereby neutralizing pro‐inflammatory signals and restoring immunological homeostasis before they can propagate systemic tissue damage [67].

DAMP detection is dependent on receptor expression. Genetic variation in pattern recognition receptors establishes baseline sensing thresholds: loss‐of‐function mutations compromising DAMP recognition (such as *TLR3* defects that disrupt antiviral DAMP detection) increase susceptibility to herpes simplex encephalitis [68]. Conversely, *TLR7* gene duplications confer excessive sensitivity to self‐derived DAMPs, precipitating pediatric systemic lupus erythematosus (SLE) and underscoring the need to maintain appropriate recognition thresholds [68].

## 3 Diverse Biological Roles of Danger Signals

As primary danger signals, PAMPs and DAMPs exhibit remarkable functional diversity, ranging from the rapid initiation of inflammatory cascades to the subtle orchestration of tissue repair. While innate immune cells are traditionally viewed as the principal decoders of these signals, it is now clear that their impact extends to adaptive lymphocytes and diverse stromal populations, which actively shape the danger response. Furthermore, emerging research is increasingly focused on the systemic effects of these molecules.

A question that often confuses researchers is how the same PAMP or DAMP can elicit divergent, and sometimes opposing, biological outcomes. To resolve this ambiguity, it is essential to recognize that the biological outcome of danger signaling is not intrinsic to the molecule itself but is fundamentally dictated by the molecular and spatiotemporal context. Factors such as molecular isoforms governed by posttranslational modifications (e.g., the redox state of HMGB1 [69]) and the synergistic cross‐talk between coincident PAMP and DAMP exposure [70] determine whether a signal promotes protective immunity or fuels maladaptive pathology.

**FIGURE 3:**
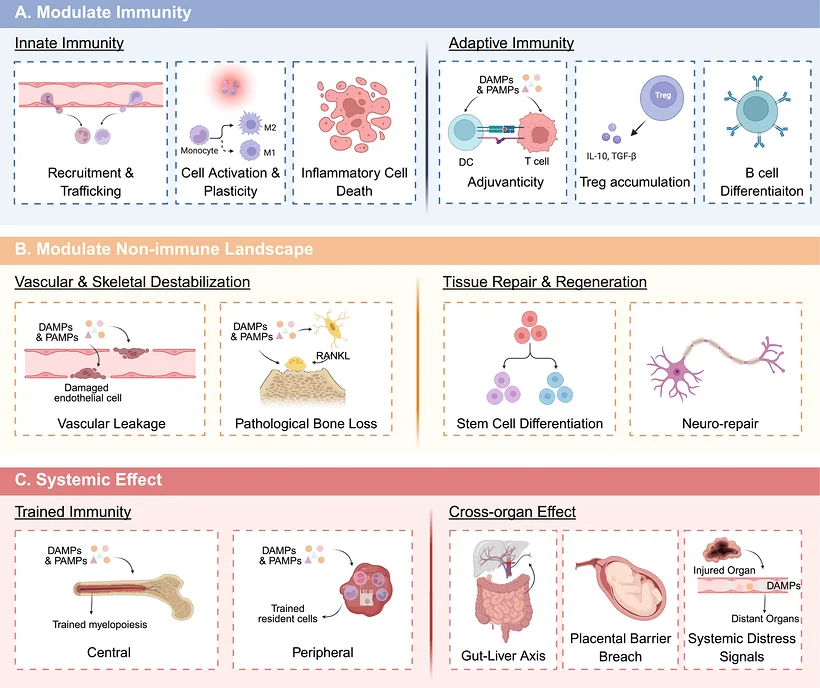
The Multifaceted roles of PAMPs and DAMPs in immunity, tissue homeostasis, and systemic integration. (A) Danger signals orchestrate immune responses by regulating leukocyte trafficking, cellular activation and plasticity, and multiple inflammatory cell death pathways. In adaptive immunity, PAMPs and DAMPs provide adjuvanticity to license DC maturation, T cell differentiation, and Treg accumulation, while also directly modulating B cell responses. (B) Beyond immune modulation, these signals reshape the nonimmune microenvironment through vascular endothelial destabilization leading to leakage, pathological bone loss via osteoclast activation, and conversely, promote tissue repair by inducing stem cell differentiation and neuro‐reparative programs. (C) At the systemic level, PAMPs and DAMPs induce trained immunity through central (bone marrow) and peripheral reprogramming, and mediate cross‐organ communication by translocating from injured tissues to distant sites via various anatomical routes, including the gut‐liver axis and placental barrier, thereby propagating systemic distress signals.

This section highlights the universal mechanisms by which these molecules modulate host defense and stromal activities, emphasizing how these signal receivers transform ancient danger signatures into specialized biological instructions (Figure 3).

### 3.1 Orchestrating the Immune Landscape

#### 3.1.1 Innate Immunity

PAMPs and DAMPs serve as primary drivers of leukocyte trafficking, coordinating the migration of immune cells from the vasculature to sites of infection or injury [71, 72]. These molecules function as direct chemoattractants. For instance, extracellular ATP released from damaged tissue acts as a canonical “find‐me” signal for myeloid cells [73]. Beyond acting as direct gradients, they activate tissue‐resident cells to release chemoattractive mediators. In schistosomiasis, DAMPs trigger the cross‐talk between P2Y2 and P2×7 purinergic receptors in endothelial cells, upregulating VCAM‐1 through NF‐κB signaling and thereby facilitating mononuclear cell adhesion [74]. Similarly, the bacterial metabolite ADP‐heptose engages the host sensor ALPK1, triggering a TIFA‐dependent cascade that induces robust chemokine secretion from epithelial cells [75]. Beyond local recruitment, these signals also govern systemic cell mobilization. The NLRP3‐caspase‐1 axis facilitates the egress of hematopoietic stem and progenitor cells (HSPCs) from the bone marrow by activating the complement cascade through a cocktail of DAMPs that includes HMGB1 and S100A9 [76].

Following recruitment, DAMPs and PAMPs amplify the inflammatory response by inducing cellular activation and phenotypic shifts. This process establishes a pro‐inflammatory state characterized by the release of reactive oxygen species (ROS) and cytokines like TNF‐α and IL‐8, as observed in neutrophils treated with DAMPs (e.g., methemoglobin) [77]. In myeloid cells, retroviral replication intermediates, such as intron‐containing HIV‐1 RNA, act as PAMPs that bypass traditional receptors to bind NLRP1, driving IL‐1β secretion [78]. These molecular gradients also dictate macrophage functional plasticity, utilizing metabolic and epigenetic reprogramming to steer the transition between pro‐inflammatory M1 and pro‐resolution M2 phenotypes [79]. In addition, the role of these danger signals is increasingly recognized in innate lymphoid cells (ILCs), which serve as a critical functional bridge between innate sensing and adaptive execution. NK cells, for instance, function as autonomous sensors of PAMPs. In the absence of accessory cells, specific bacterial PAMPs can trigger NK cells to rapidly secrete IFN‐γ and release preformed alpha‐defensins, cationic peptides that directly disrupt microbial membranes [41]. This direct activation is further exemplified by the fungal PAMP β‐1,3‐glucan, which engages NKp30 to initiate a Src family kinase‐dependent signaling cascade, leading to the polarized release of cytolytic granules containing perforin [80].

The amplification cascade intensifies when DAMPs and PAMPs trigger regulated cell death pathways, thereby eliminating infected or damaged cells [81]. Apoptosis, traditionally considered immunologically silent, can become inflammatory under certain contexts. For example, PAMPs accelerate neuron apoptosis in the presence of amyloid pathology, thereby amplifying neuron inflammation [82]. Necroptosis represents a more overtly inflammatory death pathway, exemplified by viral Z‐form RNA activating the ZBP1/RIPK3/MLKL axis to eliminate infected cells [83]. Furthermore, PAMP and DAMP signaling can initiate other specialized inflammatory cell death modalities: NETosis induced by *Candida albicans* traps and neutralizes extracellular pathogens [84, 85], whereas pyroptosis facilitates defense responses against intracellular infection [86]. PANoptosis represents the most complex integration of death pathways, in which simultaneous inflammasome activation by multiple ligands (e.g., PAMP/heme or heme/cytokine combinations) triggers multiple forms of programmed inflammatory cell death [87]. In this context, NLRP3, AIM2, NLRC4, and Pyrin assemble into a large multiprotein complex alongside ASC, caspase‐1, caspase‐8, and RIPK3 that drives PANoptosis, ensuring robust host defense against pathogens like HSV‐1 that might otherwise evade individual death pathways [88].

#### 3.1.2 Adaptive Immunity

Beyond their well‐established roles in innate activation, PAMPs and DAMPs exert a profound influence on adaptive immunity by directly modulating lymphocyte activity and restructuring the microenvironment that governs antigen‐specific responses.

The transition from a transient innate response to sustained adaptive surveillance requires more than just antigen recognition. It necessitates “adjuvanticity,” a quality primarily provided by the PAMP/DAMP axis. While antigenicity provides specificity, danger signals provide the requisite “second signals” for optimal immunogenicity. These signals drive DC maturation and enhance antigen cross‐presentation, thereby licensing the initiation of CD8^+^ T cell‐mediated antitumor or antipathogen immunity [89]. Furthermore, DAMPs such as HMGB1 can be released by DCs into the immunological synapse, where they bind to RAGE on T cells to stabilize the DC‐T cell interface and lower the threshold for TCR activation [90].

Once primed, T cell differentiation is further fine‐tuned by the local DAMP landscape. In chronic infections, the DAMP IL‐33 counterbalances type I interferon effects to prevent premature exhaustion of Tcf‐1^+^ progenitor cells, thereby ensuring sustained differentiation of functional CD8^+^ T cells [91]. Conversely, during the oral wound‐healing process, the IL‐33/ST2 axis exhibits a pro‐reparative role; ST2 is preferentially expressed on regulatory T cells (Tregs), where IL‐33 signaling promotes Treg accumulation to quench excessive inflammation and accelerate tissue closure [92]. Beyond individual cell modulation, persistent IL‐33 signaling acts as a potent driver of tertiary lymphoid structures (TLSs) in chronic inflammatory contexts such as colitis, facilitating localized immune aggregates that bridge the gap between acute damage and long‐term surveillance [93].

Within the tumor microenvironment, the impact of DAMPs is characterized by a striking duality. On one hand, IL‐33 can orchestrate potent anti‐tumor CD4^+^ and CD8^+^ T cell responses through annexin A1 signaling, and its presence can enhance the efficacy of chemotherapies like 5‐fluorouracil by priming the T cell compartment [94]. On the other hand, certain danger signals foster immune evasion. For instance, the release of the DAMP IL‐1α during tumor necroptosis can paradoxically impair myeloid cell function, subsequently suppressing CD8^+^ T cell recruitment and fostering an immunosuppressive milieu that facilitates malignancy [95].

Finally, DAMPs directly engage the humoral arm of adaptive immunity. Following myocardial infarction, heart‐derived DAMPs such as HMGB1 and HSP60 activate B cells via Toll‐like receptor signaling, promoting their rapid differentiation into antibody‐producing plasma cells [43]. This direct linkage ensures that the humoral system is mobilized not only against foreign pathogens but also in response to the molecular signatures of structural tissue failure, facilitating a comprehensive systemic defense [43].

### 3.2 Modulation of the Nonimmune Microenvironment

Beyond their immunomodulatory roles, DAMPs and PAMPs function as potent bioactive cues that reshape the nonimmune landscape, directly influencing vascular integrity, tissue remodeling, and structural homeostasis across multiple organ systems.

Vascular leakage has emerged as a critical pathological feature during circulatory failure, triggered by the inflammatory response following recognition of both PAMPs and DAMPs [96]. This hyperpermeability exacerbates circulatory collapse through hypovolemia and contributes to secondary microcirculatory disorders and organ dysfunction via interstitial edema formation. At the mechanistic level, PAMPs and DAMPs activate multiple pathways in microvascular endothelial cells, shifting them from a quiescent, barrier‐stabilized phenotype to an activated, hyperpermeable state. This transition involves destabilization of adherens and tight junctions, particularly through Src and RhoA kinase‐mediated phosphorylation and endocytosis of VE‐cadherin, alongside glycocalyx degradation, oxidative stress, and endothelial cell death [96]. Viral products further compromise endothelial integrity; for instance, treatment with NS1 alone disrupts endothelial cell monolayer integrity in an in vitro model of vascular leak, demonstrating how pathogen‐derived factors directly undermine barrier function [97]. Beyond acute permeability changes, DAMPs contribute to chronic vascular remodeling. Calprotectin (S100A8/A9) has been identified as a contributor to vascular calcification in chronic kidney disease, representing a potential therapeutic target for this complication [98].

The skeletal microenvironment is particularly sensitive to PAMP and DAMP signaling, which can drive pathological bone loss through multiple mechanisms. While traditional views focused on immune cell‐mediated bone loss, recent evidence reveals that matrix‐embedded osteocytes act as primary “sentinels” that sense bacterial PAMPs through a MYD88‐regulated signaling pathway [44]. Upon PAMP recognition, osteocytes produce significantly higher titers of the pro‐osteoclastogenic cytokine RANKL compared with osteoblasts [44]. Mechanistically, the activation of the ERK‐CREB/STAT3 signaling axis increases the binding of these transcription factors to *Rankl* enhancers. Simultaneously, DAMP/PAMP signaling suppresses the K48‐ubiquitination of CREB and STAT3, preventing their degradation and ensuring sustained high expression of *Rankl* [44]. Furthermore, virulence lipids derived from *Porphyromonas gingivalis* (a key periodontitis pathogen) can accelerate osteoclastogenesis independently of canonical HMGB1 signaling, highlighting the diversity of PAMP‐driven bone destruction [99]. This “danger signal‐bone” axis is also evident in sterile contexts. For example, osteocyte necrosis triggered by bone fracture results in excessive DAMP release. These DAMPs bind to Mincle on macrophages to induce osteoclast differentiation [100].

In contrast to their destructive roles, PAMPs and DAMPs are indispensable for initiating wound healing and tissue reparative programs. A striking example is the ALPK1‐TIFA signaling axis in the intestinal epithelium [45]. The PAMP ADP‐heptose triggers NF‐kB signaling, which initially induces TNF‐dependent apoptosis in damaged intestinal stem cells. However, this acute injury signal subsequently activates a TGF‐β and YAP‐based resurgence stem cell program. This process drives the dedifferentiation of Paneth cells into multipotent stem cells, effectively rebuilding the epithelial barrier. In models of radiation‐induced injury or DSS‐induced colitis, the absence of this PAMP‐sensing axis leads to severely impaired regenerative capacity. Similar reparative logic applies to the central nervous system, where specific DAMPs liberated poststroke coordinate the transition from acute neuroinflammation to long‐term neuronal repair and functional recovery [101].

### 3.3 Systemic and Cross‐Organ Effect

The influence of PAMPs and DAMPs is not confined to the site of initial insult; rather, these molecules serve as systemic messengers that reprogram the host immune rheostat and mediate complex interorgan communication. This integration occurs through the induction of innate immune memory and the translocation of danger signals across anatomical barriers, leading to long‐term functional shifts or distal organ dysfunction.

#### 3.3.1 Trained Immunity

Trained immunity represents a form of innate immune memory in which prior exposure to microbial products or endogenous danger signals induces long‐term functional reprogramming of innate immune cells and their progenitors through sustained metabolic and epigenetic modifications [102]. Evolutionarily, trained immunity has emerged as a beneficial host defense mechanism that provides nonspecific protection against subsequent heterologous infections. However, its dysregulation can precipitate maladaptive outcomes, fueling the pathogenesis of autoimmune disorders and chronic inflammatory diseases [103].

The role of PAMPs in inducing trained immunity has been extensively characterized across both central and peripheral compartments. Centrally, the administration of β‐glucan, a prototypical trained‐immunity‐inducing agonist, drives the expansion of myeloid lineage progenitors in the bone marrow. This process is orchestrated by elevated signaling of cytokines such as IL‐1β and GM‐CSF, alongside metabolic adaptations in glucose utilization and cholesterol biosynthesis [104]. In the periphery, LPS‐induced innate memory in airway‐resident macrophages confers robust protection against acute challenges, such as *pneumococcal* infection, without altering the stability of the resident macrophage pool [105]. Remarkably, the paradigm of trained immunity has recently expanded to include nonimmune cells. Viral PAMPs, such as poly(I:C), can induce an “epigenetic signature” in airway epithelial cells, leading to exaggerated IL‐6 release, which is critically associated with exacerbation in experimental and clinical asthma [106].

While microbial motifs were the first identified trainers, accumulating evidence confirms that endogenous DAMPs play an equally pivotal role [107]. For instance, heme has been identified as a potent inducer of long‐term trained immunity in myeloid cells. Mechanistically, heme exposure leads to enrichment of H3K27ac (histone H3 lysine 27 acetylation) at the promoters of pro‐inflammatory genes such as *TNF* and *IL‐8* [108]. This epigenetic remodeling is accompanied by the calibrated activation of the Syk and JNK pathways, thereby lowering the threshold for subsequent inflammatory responses.

Collectively, these findings position trained immunity as a central mechanism through which PAMPs and DAMPs exert prolonged effects on host defense and inflammatory pathology. The capacity to induce innate immune memory, whether protective or pathogenic, represents a fundamental dimension of PAMP/DAMP biology with profound implications for cancer immunotherapy and the management of chronic inflammatory diseases.

#### 3.3.2 Cross‐Organ Effect

The pathogenesis of inflammatory disorders frequently involves the coordinated dysfunction of multiple organ systems, in which primary damage to local tissue can precipitate concomitant pathologies at distant anatomical sites. A critical question in systemic biology is how this interorgan communication is sustained. Emerging evidence positions PAMPs and DAMPs as central molecular vehicles that facilitate this “organ crosstalk” by traversing physiological barriers and disseminating inflammatory signals through the systemic circulation [9].

PAMPs are no longer viewed as merely localized infectious markers but as systemic modulators that can redefine the health of remote organs. Under conditions of intestinal dysbiosis, gut‐derived PAMPs translocate across the compromised epithelial barrier into the portal circulation and eventually reach the liver. Here, the intracellular sensor NOD2 recognizes muramyl dipeptide (MDP), a PAMP motif present in both Gram‐positive and Gram‐negative bacteria. This sensing mechanism translates gut‐derived signals into pro‐tumorigenic pathways in the liver, significantly contributing to hepatocarcinogenesis [109]. Perhaps more striking is the ability of PAMPs to bypass the placental barrier. Bacterial cell wall peptidoglycan (CW), a universal ligand for TLR2, has been shown to traverse the murine placenta and enter the developing fetal brain. This systemic translocation activates the CW‐TLR2 signaling axis within the fetal neuroenvironment, potentially disrupting neurodevelopment and predisposing the offspring to postnatal cognitive and behavioral disorders [110].

In addition to PAMPs, endogenous DAMPs released from necrotic or ischemic tissues also serve as potent systemic messengers of distress, often driving lethal systemic inflammation and multiple organ dysfunction syndrome (MODS). Following severe trauma or tissue injury, cell‐free mtDNA is liberated from the damaged parenchyma into the systemic circulation. Once released in the blood, mtDNA activates circulating neutrophils by inducing rapid Ca2^+^ signaling and p38 MAPK phosphorylation. This molecular activation drives neutrophil chemotaxis and degranulation at remote sites, such as the lungs and liver, leading to neutrophil‐mediated organ injury that mimics the inflammatory profile of sepsis [111]. Similarly, in skeletal muscle I/R injury, the DAMP HMGB1 translocates from the nucleus to the cytoplasm and is subsequently released into the systemic circulation upon reperfusion. This circulating HMGB1 serves as a long‐range signal that communicates the local ischemic insult to the entire host, potentially triggering secondary inflammatory cascades in distal organs [112].

Collectively, these systemic and cross‐organ integration mechanisms underscore that PAMPs and DAMPs are not merely local byproducts of injury, but are active participants in the global regulation of host physiology and the systemic progression of disease.

### 3.4 Interplay Between PAMPs and DAMPs

The host immune system operates within a complex molecular environment where exogenous microbial products and endogenous damage signals coexist. The interplay between PAMPs and DAMPs encompasses a spectrum of interactions ranging from synergistic amplification to mutual antagonism, collectively shaping inflammatory outcomes through distinct mechanistic pathways. Understanding this dynamic crosstalk is essential for deciphering the pathophysiology of conditions like sepsis and for designing rational therapeutic interventions that account for the integrated danger context rather than targeting individual signals in isolation.

#### 3.4.1 Synergistic Amplification of Inflammation

Research has revealed that the concurrent presence of PAMPs and DAMPs frequently triggers inflammatory responses that exceed the sum of individual stimuli, with the nature and magnitude of synergy depending critically on the specific molecular context. In some cases, DAMPs alone are insufficient to activate inflammation but become potent amplifiers in the presence of microbial products. For example, while low‐dose extracellular RNA alone does not promote the pro‐inflammatory activation of astrocytes, it induces robust cytokine expression when combined with the synthetic PAMP molecule Pam2CSK4 [70]. Alternatively, DAMPs with intrinsic stimulatory capacity can have their effects markedly amplified by concomitant PAMP exposure, as seen when methemoglobin‐induced production of ROS and pro‐inflammatory cytokines in neutrophils is further intensified by the addition of the LTA complex [77].

This cooperative behavior operates through at least two distinct mechanistic pathways. One major mechanism involves transcriptional reprogramming, in which DAMP‐PAMP co‐stimulation activates transcription factors that upregulate pattern recognition receptor expression, creating a feed‐forward amplification loop. A primary example is the DAMP heme, released during hemolysis, which synergizes with LPS via TLR2/4‐dependent IRF1 activation; this process leads to the transcriptional upregulation of NLRP12, which qualitatively alters gene expression to prime cells for heightened inflammasome activity [113]. Beyond transcriptional reprogramming, DAMPs and PAMPs also collaborate through threshold modulation, wherein endogenous DAMPs lower the detection threshold for microbial products. Extracellular HSP60 binds LPS and facilitates microbe recognition by reducing the threshold for PAMP detection and enhancing TLR signaling, effectively functioning as a molecular bridge that sensitizes the innate immune system to concomitant microbial challenges [114].

#### 3.4.2 Inhibitory and Regulatory Interactions

Paradoxically, DAMP‐PAMP interactions are not invariably pro‐inflammatory; under specific contexts, endogenous damage signals exert inhibitory effects that restrain microbial product‐driven inflammation, revealing built‐in regulatory circuits that prevent excessive immune activation and preserve tissue integrity. Receptor desensitization through internalization is a major inhibitory mechanism in which DAMPs induce the endocytosis of PAMP receptors, rendering cells hyporesponsive to subsequent microbial stimulation. In a mouse model simulating long‐bone fracture, LPS promotes macrophage necroptosis through TLR4 signaling, yet this PAMP‐driven cell death is ameliorated by HMGB1 released from damaged tissue [115]. Mechanistically, HMGB1 engages RAGE, upregulating caveolin‐1 expression through RAGE‐MyD88‐dependent Cdc42 activation and subsequent recruitment of the transcription factor Sp1, leading to caveolae‐mediated TLR4 internalization and desensitization, thereby reducing macrophage necroptosis. Another distinct regulatory pathway involves independent priming defense, wherein DAMPs can provide an independent priming defense that restricts pathogen replication before the canonical PAMP‐mediated interferon response is fully mounted. Oxidized lipids exemplify this protective dimension [116]. Exposure of cells to these DAMPs prior to vesicular stomatitis virus infection triggers a rapid, interferon‐independent antiviral state by preventing viral entry and restricting the percentage of productively infected cells, thereby serving as an early homeostatic brake that limits viral load during the nascent stages of PAMP‐mediated viral inferno responses.

## 4 Danger Signals Across Human Diseases

**FIGURE 4:**
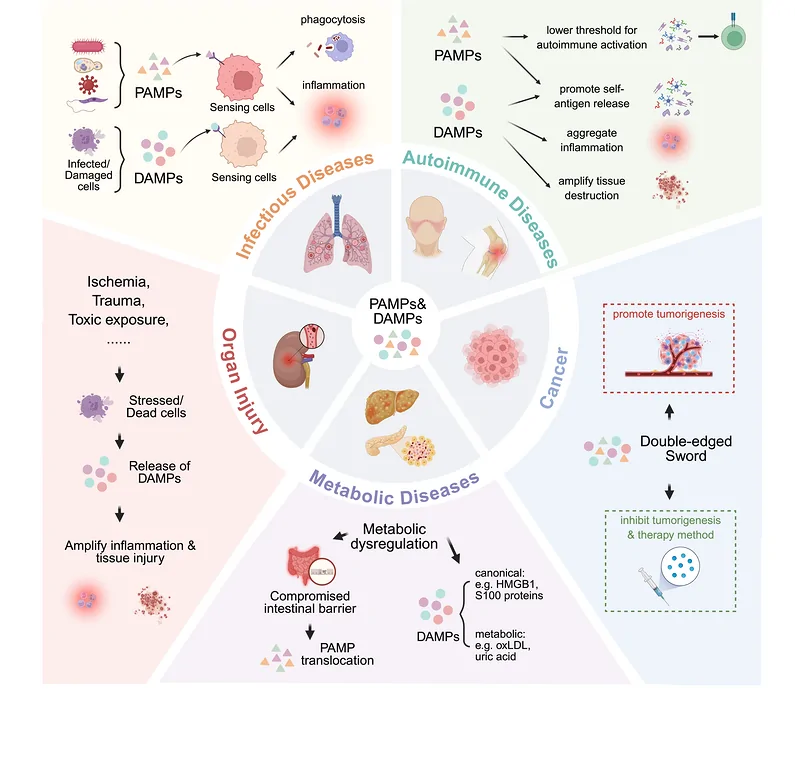
The pathological role of PAMPs and DAMPs in human diseases. PAMPs and DAMPs function as central orchestrators of disease pathogenesis through five distinct modalities. In infectious diseases, PAMPs and DAMPs trigger innate sentinels to initiate phagocytosis and inflammatory responses. In autoimmune disease, PAMPs and DAMPs function as critical amplifiers that provide autoantigen sources, propagate inflammation, and sustain chronic tissue injury. In the context of malignancy, danger signals exhibit a “double‐edged sword” duality, either fueling tumorigenesis through chronic inflammation or inhibiting it by facilitating immunogenic cell death and therapeutic responses. During organ injury, stressors such as ischemia and trauma induce the release of endogenous alarmins from dying cells, establishing pro‐inflammatory feed‐forward loops that exacerbate secondary tissue injury. In metabolic disorders, pathogenesis involves the translocation of gut‐derived PAMPs across compromised barriers and the accumulation of DAMPs following metabolic dysregulation.

The preceding sections have established the molecular identities, sensing mechanisms, and regulatory networks that govern PAMP and DAMP signaling under homeostatic and pathological conditions. Building upon this foundation, we now examine how these danger signals integrate into the complex pathology of human disease, including infectious disease, autoimmune disorder, malignancy, organ injury, and metabolic disease (Figure 4).

### 4.1 Infectious Disease

Infectious diseases, despite their diverse clinical manifestations, converge on a fundamental pathophysiological principle: the host immune system's recognition of microbial non‐self‐initiates a cascade of events that must be precisely calibrated to eliminate pathogens while preserving tissue integrity. This recognition is primarily mediated by PAMPs, which serve as the requisite “first signal” for acute inflammatory activation [117]. However, as infection progresses, collateral tissue injury and cellular stress trigger the liberation of endogenous DAMPs, establishing a dynamic interplay in which exogenous and endogenous danger signals collectively shape disease outcomes [118].

The PAMP/DAMP sensing machinery plays an indispensable role in antimicrobial defense across diverse pathogens. In bacterial infections, the ALPK1‐TIFA signaling axis exemplifies a critical gatekeeper of pulmonary immunity; *Burkholderia cenocepacia* infection models demonstrate that ALPK1 (a PRR) deficiency markedly attenuates lung inflammation but simultaneously permits uncontrolled bacterial proliferation, underscoring the necessity of this PAMP pathway for effective host defense [14]. Among viral sensors, MDA5 deficiency similarly increases susceptibility to severe infections, highlighting the nonredundant functions of cytosolic PRRs in antiviral immunity [119]. These examples collectively illustrate that PAMP/DAMP sensing constitutes an essential first line of defense, without which pathogen containment fails.

However, the PAMP/DAMP signaling axes that drive protective immunity can also precipitate catastrophic pathology when dysregulated. For example, the NS1 protein of dengue virus mimics LPS to disrupt endothelial integrity and contribute to life‐threatening vascular leakage by engaging TLR4 [97]. Sepsis represents the ultimate manifestation of such dysregulation, in which the interplay among PAMPs, DAMPs, and inflammatory cell death pathways spirals into a self‐perpetuating systemic inflammatory response [120]. Circulating DAMPs, including extracellular HMGB1 and histones, drive concurrent activation of complement and coagulation cascades, leading to disseminated intravascular coagulation and multiple organ dysfunction syndrome [121]. This transition from protective immunity to immunopathology illustrates the double‐edged nature of danger signaling: the very mechanisms evolved to eliminate pathogens can, when overwhelming or sustained, become primary drivers of tissue destruction and mortality.

### 4.2 Autoimmune Diseases

Autoimmune diseases arise from the breakdown of immunological tolerance, wherein genetic susceptibility and environmental triggers converge to unleash autoreactive lymphocytes against self‐tissues, resulting in chronic inflammation and tissue injury [122]. Within this pathogenic cascade, PAMPs and DAMPs function as critical amplifiers that provide autoantigen sources, propagate inflammation, and sustain the chronic tissue injury characteristic of these disorders.

Genetic susceptibility establishes the baseline threshold for immune dysregulation. Recent evidence highlights genetic dysregulation in DAMP/PAMP‐sensing receptors as a pivotal driver of autoimmunity. Excessive TLR7 (a classic DAMP sensor) signaling triggered by self‐nucleic acid recognition has been implicated in SLE pathogenesis. Cells from patients harboring the UNC93B1 E92G variant produce elevated levels of TNF‐α and IL‐6 upon TLR7/TLR8 agonist stimulation. This E92G mutation destabilizes the UNC93B1 protein and attenuates its interaction with TLR7, leading to selective TLR7 hyperactivation accompanied by constitutive type I interferon signaling that accelerates SLE progression [123].

Microbial PAMPs serve as potent environmental catalysts that lower the threshold for autoimmune activation. For example, PAMP (such as the Epstein‐Barr virus nuclear antigen‐1) exhibits cross‐reactivity with lupus‐associated self‐antigens, leading the immune system to inadvertently target host tissues [124]. Furthermore, through engagement of pattern recognition receptors, PAMPs enhance antigen presentation and costimulatory signals, effectively providing the adjuvant activity required to break self‐tolerance [122].

DAMPs and PAMPs promote the release of autoantigens. For instance, these molecules trigger NETosis, which results in the uncontrolled release of neutrophil extracellular traps that externalize intracellular structures, serving as a concentrated source of autoantigens [125]. DAMPs themselves can also function as integral components of immunostimulatory complexes. HMGB1 released from apoptotic cells assembles into complexes with dsDNA fragments that activate autoreactive B cells [126]. The progression toward clinical autoimmunity is also fueled by the DAMP‐mediated impairment of apoptotic cell sequestration. In SLE, HMGB1 masks phosphatidylserine on the surface of dying cells, effectively blocking their recognition by phagocytes. This impaired sequestration leads to secondary necrosis, releasing nucleosomes and mtDNA that function as primary autoantigens [127].

Additionally, extensive evidence demonstrates that DAMPs amplify inflammation in autoimmune disease through receptor engagement [128, 129]. In antigen‐induced arthritis (AIA), S100A8/A9 binds Toll‐like receptors on macrophages, promoting pro‐inflammatory cytokine production [130]. Conversely, recent evidence indicates that S100A8/A9 can mitigate the severity of autoimmune arthritis by promoting the expansion and activation of myeloid‐derived suppressor cells (MDSCs) [131]. This functional dichotomy appears to be dictated by the duration of alarmin exposure in a temporally‐dependent manner. Transient stimulation of immature bone marrow‐derived dendritic cells (BMDCs) with S100A8/A9 fosters pro‐inflammatory activity. In contrast, prolonged exposure of early‐stage myeloid progenitors to DAMPs skews their development toward an immunosuppressive lineage [131]. These findings underscore the profound functional plasticity of DAMPs, in which the biological outcome is determined not only by the ligand‐receptor pair but also by the chronicity of the stimulus within the microenvironment.

Beyond immune modulation, DAMPs directly amplify tissue destruction in autoimmune disease. In arthritic contexts, S100A8/A9 stimulates chondrocytes to upregulate matrix metalloproteinases, culminating in the targeted degradation of cartilage matrix [132].

### 4.3 Cancer

The role of PAMPs and DAMPs in oncology is defined by a complex duality. These molecules act as double‐edged swords in malignancy, as they can either facilitate tumor growth and immune evasion, or orchestrate robust antitumor immunity [133].

Chronic liberation of DAMPs and PAMPs within established tumors often fosters an inflammatory environment that supports cancer progression. Persistent danger signaling polarizes the immune landscape toward suppression. For example, HMGB1 facilitates the recruitment and accumulation of MDSCs while concurrently inhibiting the cytotoxic function of CD8^+^ T cells [134, 135]. Similarly, the S100A8/A9 signaling axis facilitates tumor malignancy by driving the expansion of aberrant granulocyte‐monocyte progenitors and skewing bone marrow hematopoiesis toward a myeloid bias, which ultimately impairs T cell‐mediated anti‐tumor surveillance [136]. Intratumoral *F. nucleatum* drives gastric cancer immune evasion by triggering the NF‐κB signaling axis and the subsequent recruitment of tumor‐associated neutrophils [137]. Danger signals directly affect tumor and stromal cells, promoting survival and spread. In conditions like myeloproliferative neoplasms, malignant S100A8/A9 release activates TLR4 and RAGE on mesenchymal stromal cells, inducing their transformation into pro‐fibrotic myofibroblasts that create a protective survival niche for leukemic cells [138].

Conversely, when appropriately engaged, often through therapeutic intervention, DAMPs and PAMPs are essential for initiating protective anti‐tumor responses. The concept of immunogenic cell death (ICD) illustrates how danger signals license adaptive immunity [139]. Tumor cells frequently subvert immunosurveillance by limiting the release of ICD‐associated molecular patterns; specifically, the upregulation of the ENTPD1/CD39 ectonucleotidase axis by tumor cells facilitates the enzymatic depletion of extracellular ATP, thereby hampering the chemotactic recruitment of immune effectors [140]. Adynerin‐mediated induction of ICD in breast cancer cells, characterized by the surface translocation of calreticulin and the liberation of HMGB1, HSP70/90, and ATP, effectively primes the phenotypic maturation and activation of dendritic cells, ultimately augmenting the cytotoxic potency of CD8^+^ T lymphocytes [141]. Through ICD, dying tumor cells are effectively converted into “endogenous vaccines,” shifting the parenchymal state from an immunosuppressive source to a primary driver of T cell priming. In addition, DAMPs and PAMPs can induce “trained immunity”, an epigenetic and metabolic reprogramming of innate cells that enhances their long‐term antitumor responsiveness [142]. Exploiting these pathways has led to the development of potent immunotherapies. For instance, BCG engineered to overexpress the STING‐pathway activator c‐di‐AMP, one type of PAMP, demonstrates superior efficacy by amplifying the epigenetic modifications required for sustained anti‐tumor vigilance [143].

### 4.4 Organ Injury

Sterile organ injury represents a pathological continuum wherein initial tissue damage, whether from ischemia, trauma, or toxic exposure, triggers DAMP release that both signals the initial damage and amplifies subsequent tissue destruction through feed‐forward inflammatory loops. This self‐propagating mechanism explains why the severity of organ injury often exceeds what would be predicted from the initial insult alone and why limiting DAMP‐mediated amplification represents a critical therapeutic opportunity.

Ischemia‐reperfusion (I/R) injury provides a typical model for this DAMP‐driven destruction. While the ischemic phase induces profound tissue hypoxia, the subsequent restoration of blood flow paradoxically exacerbates cellular demise, a phenomenon with severe clinical implications across multiple organ systems [144]. Mechanistic studies have elucidated diverse organ‐specific DAMP pathways. In the liver, I/R challenges suppress the expression of HSPA12A, which subsequently facilitates glycolysis‐mediated HMGB1 secretion. This DAMP acts as a potent driver of macrophage chemotaxis and activation, intensifying hepatic tissue damage [145]. Within the kidney, I/R triggers the release of Peroxiredoxin 1 (Prdx1). Functioning as a canonical DAMP, Prdx1 engages the Mincle/Syk/NF‐κB signaling axis to amplify the inflammatory cascade and exacerbate acute kidney injury [146]. In ocular contexts, sudden elevations in intraocular pressure induce retinal damage and the liberation of double‐stranded DNA. This nucleic acid DAMP activates the cGAS‐STING pathway, initiating the inflammatory responses that drive the progression of acute glaucoma [147].

### 4.5 Metabolic Disease

Metabolic diseases, such as nonalcoholic fatty liver (NAFLD) and type 2 diabetes, represent a systemic inflammatory state where nutritional excess triggers a self‐perpetuating cycle of immunological alarm [148]. This pathological landscape is initiated by the metabolic environment itself; for instance, adipose expansion and nutrient overload compromise the intestinal barrier, leading to gut dysbiosis and the systemic translocation of microbial PAMPs [149]. Simultaneously, metabolic stress promotes a dual‐layered DAMP response: canonical alarmins are liberated through accelerated cell death, while specialized “metabolic DAMPs”, including oxLDL, uric acid, and cholesterol crystals, are generated as direct byproducts of nutrient dysregulation [71]. Once liberated, these PAMPs and DAMPs act as potent drivers of disease progression by engaging innate pattern‐recognition receptors, fueling chronic, low‐grade inflammation.

The progression from NAFLD to nonalcoholic steatohepatitis (NASH) is orchestrated by a synergistic “multi‐hit” interplay between gut‐derived PAMPs and hepatocyte‐derived DAMPs, which transform the hepatic microenvironment into a chronic inflammatory niche [150]. Chronic high‐fat intake and sedentary lifestyles drive systemic insulin resistance and intestinal dysbiosis, compromising the gut barrier and facilitating the translocation of microbial LPS into the portal circulation [149]. Upon reaching the liver, these PAMPs engage TLR4 on Kupffer cells and infiltrating monocytes, providing the primary priming signal for the NLRP3 inflammasome [151]. Concurrently, excessive free fatty acid flux induces lipotoxicity, mitochondrial oxidative stress, and subsequent hepatocyte demise via apoptosis, necroptosis, or pyroptosis [152]. This cellular injury triggers the massive liberation of endogenous DAMPs, including HMGB1, mtDNA, ATP, and cholesterol crystals, which amplify local inflammation [150]. These signals further establish a self‐perpetuating feedback loop by affecting neighboring cholesterol‐loaded hepatocytes in a paracrine way [153]. Ultimately, the convergence of these danger signals sustains an unresolved inflammatory milieu that promotes collagen deposition, vascular remodeling, and the eventual transition to cirrhosis and hepatocellular carcinoma.

## 5 PAMPs/DAMPs as Biomarkers and Therapeutics

The clinical translation of danger signal biology focuses on utilizing these molecules as disease biomarkers and therapeutic targets. Because PAMP and DAMP levels closely reflect tissue damage and immune activation, their quantification in biofluids provides objective readouts of pathological states. Therapeutically, the dual nature of these signals necessitates context‐dependent strategies: inhibiting their activity can mitigate hyperinflammation in sterile injury and autoimmunity, while activating these pathways can help overcome immune tolerance in malignancies and chronic infections.

### 5.1 PAMPs/DAMPs as Clinical Biomarkers

Beyond their role in pathogenesis, PAMPs serve as valuable biomarkers, offering diagnostic precision, prognostic assessment, and therapeutic guidance in specific infectious contexts (Table 1). Invasive fungal infections exemplify this clinical utility: although culture remains the gold standard for diagnosis, its sensitivity is often suboptimal, and results can take days to obtain. Among nonculture‐based methods, (1→3)‐β‐D‐glucan, a conserved pan‐fungal cell wall component, has emerged as the most widely adopted biomarker, demonstrating excellent negative predictive value at an 80 pg/mL threshold in intensive care settings [154, 155]. Serum (1→3)‐β‐D‐glucan also reflects intestinal inflammation in Crohn's disease, as it translocates across compromised mucosal barriers, and thus serves as a noninvasive indicator of disease activity [156]. Bacterial PAMPs have likewise been harnessed through innovative detection platforms; for instance, polymyxin B‐based probes targeting LSP enable selective quantification of circulating outer membrane vesicles via nano‐flow cytometry, facilitating early diagnosis of bacterial infections even before blood cultures turn positive [157]. Viral PAMPs further expand the biomarker repertoire. Circulating HBV RNA, for example, provides a noninvasive surrogate for monitoring the intrahepatic transcriptional reservoir and guiding safe discontinuation of antiviral therapy in chronic hepatitis B [158, 159].

**TABLE 1:** PAMPs and DAMPs as biomarkers.

Biomarker name	Disease	Sample	Clinical utility	Detection technique	Reference
β‐D‐glucan	Nondialysis chronic kidney disease	Serum	Treatment response	ELISA	[168]
β‐D‐glucan	Invasive candidiasis	Serum	Treatment response	Chromogenic assay	[169, 170, 171]
Lipoarabinomannan	Tuberculosis	Sputum	Treatment response	ELISA	[172]
Lipopolysaccharides	Gastrointestinal discomfort	Fecal	Treatment response	ELISA	[173]
Lipopolysaccharides	Gastrointestinal syndrome	Plasma	Treatment response	ELISA	[174]
Lipopolysaccharides	Metabolic endotoxemia	Serum	Early diagnosis	ELISA	[175]
Lipopolysaccharides	Obesity	Serum	Treatment response	ELISA	[176]
Lipopolysaccharides	Coronary artery diseases	Serum	Treatment response	ELISA	[177]
Lipopolysaccharides	Gut barrier dysfunction	Serum	Treatment response	ELISA	[178, 179]
Lipopolysaccharides	Abdominal pain	Fecal	Treatment response	ELISA	[180]
Hepatitis B Virus RNA	Chronic hepatitis B	Serum	Treatment response	PCR	[159]
Hepatitis B Virus RNA	Chronic hepatitis B	Serum	Treatment response	PCR	[181]
HIV‐1 RNA	HIV‐1 infection	Plasma	Prognostic prediction, treatment response	PCR	[182]
SARS‐CoV‐2 RNA	COVID‐19	Plasma	Prognostic prediction, treatment response	PCR	[183]
HMGB1	Breast cancer	Plasma	Treatment response	ELISA	[184]
HMGB1	Systemic lupus erythematosus	Urine	Early diagnosis	Flow Cytometry	[185]
HMGB1	Sepsis	Serum	Treatment response	ELISA	[186]
HMGB1	Sepsis	Plasma & IP Fluid	Treatment response	ELISA	[187]
mtDNA	Sepsis	Plasma	Prognostic prediction	qPCR	[165]
HMGB1	Polycystic ovary syndrome (PCOS)	Blood	Early diagnosis, treatment response	ELISA	[188]
HMGB1	Pneumococcal bacteremia	Sputum	Early diagnosis	ELISA	[189]
HMGB1, HSP70, LL‐37, S100A8	COPD	Sputum	Early diagnosis	ELISA	[190]
HMGB1, S100A9, LL37	COPD	Serum	Early diagnosis	ELISA	[191]
HMGB1	Pneumonia	Serum	Prognostic prediction	ELISA	[192]
HMGB1	Lung cancer	Lung tissue	Treatment response	ELISA	[193]
HMGB1	Kidney transplantation	Serum	Prognostic prediction	ELISA	[194]
HMGB1	ARDS	Serum	Treatment response	ELISA	[195]
HMGB1	ARDS, MODS	Plasma	Prognostic prediction, treatment response	ELISA	[196]
HMGB1	Urothelial carcinoma of bladder	Serum	Early diagnosis	ELISA	[161]
HMGB1	Recurrent ovarian cancer	Plasma	Treatment response	ELISA	[197]
HMGB1	Liver fibrosis	Serum	Early diagnosis	ELISA	[198]
HMGB1	Cirrhosis	Serum	Treatment response	ELISA	[199]
HMGB1	β‐thalassemia major	Serum	Early diagnosis, prognostic prediction	ELISA	[200]
HMGB1	Parkinson's disease	Serum	Treatment response	ELISA	[201]
HMGB1	Epilepsy	Serum	Treatment response	ELISA	[202]
HMGB1	Type 2 diabetes mellitus	Serum	Treatment response	ELISA	[203]
DAMPs (HMGB1, HSP70, mtDNA)	Surgical stress	Plasma	Prognostic prediction	ELISA, qPCR	[204]
HMGB1	Leiomyosarcoma	Blood	Prognostic prediction	ELISA	[205]
HMGB1	Acute cerebral infarction	Serum	Treatment response	RT‐qPCR, ELISA	[206]
HMGB1	Colorectal disease	Plasma	Treatment response	ELISA	[207]
HMGB1	Unstable angina	Serum	Treatment response	ELISA	[208]
S100A9	Meibomian gland dysfunction‐related dry eye	Tear	Treatment response	ELISA	[209]
S100A8/9, S100A12	Polyarticular Juvenile idiopathic arthritis	Serum	Treatment response	ELISA	[210]
S100A8/A9	Atopic dermatitis	Cheek and antecubital fossa	Early diagnosis	ELISA	[211]
S100A8/A9	Periprosthetic Joint Infection	Synovial Fluid	Early diagnosis	ionization time‐of‐flight mass spectrometry	[212]
S100A8	Necrotizing soft‐tissue infections	Plasma	Early diagnosis	Luminex multiplex assays	[213]
S100A8/A9	Breast cancer	Serum	Prognostic prediction	ELISA	[214]
S100A8/A9	Acquired aplastic anemia and myelodysplastic syndromes	Plasma	Early diagnosis	ELISA	[215]
S100A8/A9	Hodgkin lymphoma	Serum	Treatment response	ELISA	[216]
S100A8/A9	Vasculitis	Serum	Prognostic prediction	ELISA	[217]
S100B	Postoperative brain injury	Serum, plasma	Prognostic prediction	ELISA	[218]
S100A12	Rheumatoid arthritis	Plasma, synovial tissue	Treatment response	ELISA, immunohistochemistry	[164]
mtDNA	Myocardial infarction	Plasma	Prognostic prediction	qPCR	[219]
mtDNA	Subarachnoid hemorrhage	Serum	Prognostic prediction	qPCR	[220]
mtDNA	Inflamm‐aging	Plasma	Prognostic prediction		[221]
mtDNA	Chronic kidney disease	Serum	Prognostic prediction	RT‐PCR	[222]
mtDNA	SIRS, MODS	Plasma	Prognostic prediction	qPCR	[223]
mtDNA	Sickle cell disease	Plasma	Prognostic prediction	qPCR	[224]
mtDNA	Postoperative pneumonia	Plasma	Prognostic prediction	qPCR	[225]
mtDNA	Colorectal cancer	Plasma	Early diagnosis	qPCR	[226]
mtDNA	Living donor kidney transplantation	Plasma	Prognostic prediction	qPCR	[227]

DAMPs have emerged as premier candidates for biomarker development, owing to their high detectability and quantifiable abundance (Table 1). As endogenously derived molecules released extracellularly, DAMPs readily accumulate in accessible biofluids such as serum, plasma, and synovial fluid, thereby facilitating minimally invasive diagnostic procedures [160]. In clinical practice, DAMPs serve as pivotal indicators for early diagnosis. For instance, HMGB1 has exhibited favorable sensitivity and specificity profiles and demonstrated significant correlations with key clinicopathological parameters, positioning it as a promising diagnostic candidate for early detection of urothelial bladder cancer [161]. In addition, DAMPs facilitate the longitudinal monitoring of disease progression. Elevated circulating S100A8/A9, for example, has been validated as a reliable marker of disease activity in ANCA‐associated vasculitis [162]. Notably, S100A8/A9 has also emerged as a powerful predictor of heart failure following acute myocardial infarction, demonstrating prognostic performance that even surpasses established biomarkers including cardiac troponin I (cTnI), B‐type natriuretic peptide (BNP), and C‐reactive protein (CRP) [163]. Furthermore, DAMPs can predict the efficacy of interventions. A progressive decline in serum S100A12 levels serves as a molecular surrogate for attenuated synovial neutrophil activation, effectively mirroring the success of anti‐inflammatory therapies such as intra‐articular corticosteroid treatment in rheumatoid arthritis [164]. Interestingly, DAMPs exhibit superior diagnostic performance compared with PAMPs in certain contexts. For instance, in early sepsis, cell‐free DNA (cfDNA) predominantly comes from host origin (∼99.86%), whereas microbial‐derived cfDNA (a PAMP) constitutes a negligible fraction (∼0.077%), suggesting that host‐derived DAMPs provide a more robust signal for early detection than pathogen‐derived markers [165].

Despite substantial progress, several methodological limitations complicate the interpretation of DAMP biology. First, conventional detection methods such as ELISA primarily quantify total protein levels and fail to distinguish functionally distinct isoforms governed by posttranslational modifications. This technical shortfall may explain the inconsistent correlations observed between total DAMP concentrations and disease progression in clinical studies [166]. While mass spectrometry remains the gold standard for characterizing posttranslational modifications, such as the critical redox states of HMGB1, its high cost and technical complexity currently render it incompatible with routine diagnostics [166]. Second, once released, certain DAMPs undergo rapid extracellular degradation. Serum HMGB1, for example, exhibits a relatively short half‐life of approximately 11 ± 1 h, with its thiol isoform decaying even more rapidly at 17 ± 1 min [167]. The rapid turnover makes it difficult for accurate diagnosis. Third, circulating DAMP concentrations may not faithfully reflect local tissue events due to dilution effects and enzymatic degradation by nucleases and proteases. DAMPs typically accumulate at high concentrations within damaged tissues, such as bone defects or cirrhotic liver parenchyma, but become substantially diluted or sequestered by clearance receptors upon entering the peripheral circulation. Consequently, peripheral blood measurements may underestimate the true burden of danger signals within the tissue microenvironment, posing a significant challenge for biomarker development and clinical translation.

### 5.2 PAMPs/DAMPs as Therapy Targets

**FIGURE 5:**
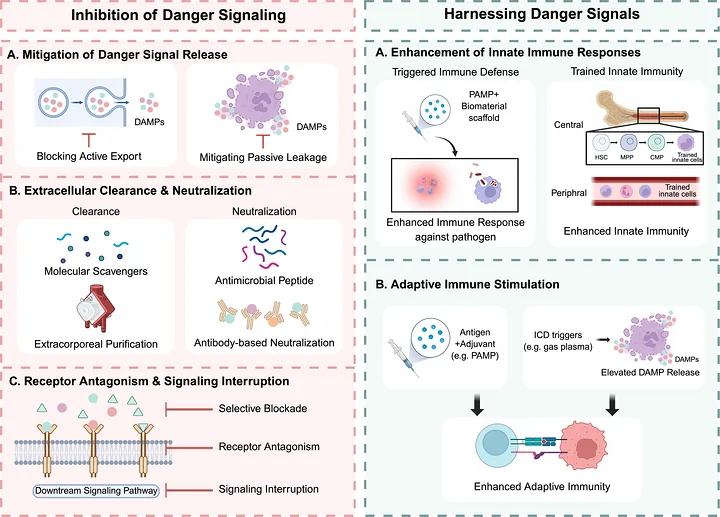
Therapeutic modalities targeting the PAMP/DAMP axis through pathological inhibition and immunological harnessing. Inhibition of danger signaling (left) involves a hierarchical approach to quench hyperinflammation: (A) mitigation of release by blocking active secretory pathways or passive membrane leakage; (B) extracellular clearance using molecular scavengers, extracorporeal purification, or antibody‐mediated neutralization; and (C) signaling interruption via selective receptor antagonism and downstream pathway blockade. Harnessing danger signals (right) utilizes these molecules as therapeutic stimuli to revitalize host defenses: (A) innate enhancement through biomaterial‐based PAMP delivery to trigger immediate defense and the induction of trained immunity via metabolic/epigenetic reprogramming of central and peripheral progenitors; and (B) adaptive stimulation by leveraging PAMPs as vaccine adjuvants or triggering immunogenic cell death (ICD) to liberate endogenous DAMPs that license robust T cell‐mediated antitumor immunity.

The PAMP/DAMP signaling axis represents a versatile therapeutic target that requires a bi‐directional modulation strategy depending on the specific immunological environment. In hyperinflammatory contexts such as sepsis or autoimmunity, clinical strategies prioritize attenuating these danger signals to quench cytokine storms and prevent systemic organ dysfunction. Conversely, in immune‐silent environments like tumors or chronic infections, therapy focuses on the strategic augmentation of PAMP/DAMP signaling to restore immunological vigilance. In recent years, several therapeutic interventions targeting these pathways have already entered clinical‐phase evaluation across diverse disease models (Table 2). In the following section, we will introduce advances in both aspects (Figure 5).

**TABLE 2:** Clinical trials targeting PAMP/DAMP pathways.

Therapeutic agent	Clinical indication	Mechanism of action	Molecular target	Clinical trial no.	Trial phase	Reference
CD24Fc	Severe COVID‐19	Neutralize HMGB1 and HSPs	HMGB1, HSPs	NCT04317040	Phase III	[228]
CD24Fc	Acute GVHD	Neutralize DAMPs	DAMPs	NCT02663622	Phase IIa	[229]
Glycyrrhizin	Progressive vitiligo	Inhibit HMGB1 release	HMGB1	ChiCTR2400085923, ChiCTR2400086844	Phase II	[230]
DSTAT + azacitidine	AML or MDS	Block HMGB1	HMGB1	NCT02995655	Pilot Study (Phase Ib/II)	[231]
Standard‐volume plasma‐exchange	Acute liver failure	Extracorporeal physical removal of endotoxin and DAMPs	Endotoxin and DAMPs	NCT02718079	Phase II (RCT)	[232]
DIALIVE	Acute‐on‐chronic liver failure	Extracorporeal removal of endotoxin, inflammasome ligands, and dysfunctional albumin.	Endotoxin, cytokeratin‐18, and inflammasome ligands	NCT03065699	Phase I (First‐in‐man)	[233]
Efferon LPS Cartridges	Sepsis	Extracorporeal physical removal of PAMPs	LPS	NCT04827407	Multicenter RCT	[234]
SM17	Allergic asthma	Blocks IL‐17RB	IL‐17RB	NCT05332834	Phase I	[235]
Selnoflast	Ulcerative colitis	NLRP3 inhibitor	NLRP3 inflammasome	ISRCTN16847938	Phase Ib	[236]
DNX‐2401	Recurrent malignant glioma	Aggregate adaptive immune response by promoting ICD‐triggered DAMPs release	Tumor cell	NCT00805376	Phase I	[237]
Doxorubicin + dacarbazine + nivolumab	Advanced leiomyosarcoma	Aggregate adaptive immune response by promoting ICD‐triggered DAMPs release	Tumor cell	NCT03277924	Phase Ib	[205]
Imprime + pembrolizumab	Metastatic TNBC	PAMP‐mediated immune activation	β‐Glucan	NCT02981303	Phase II	[238]
SpFN/ALFQ vaccine	COVID‐19	Vaccine with PAMP‐rich adjuvant to induce adaptive immunity	Viral spike protein, MPLA	NCT04784767	Phase I	[239]

#### 5.2.1 Inhibition of PAMP/DAMP Activity

Targeted inhibition of excessive PAMP/DAMP signaling represents a pivotal strategy for mitigating hyperinflammation and preventing collateral tissue damage. Current pharmacological research can be concluded at three levels: mitigating the initial release of danger signals, clearing or neutralizing PAMPs/DAMPs, and interrupting downstream receptor engagement or intracellular signaling.

The primary defensive strategy involves sequestering danger molecules within their original compartments. Preventing DAMP release is achievable by modulating either active secretion or passive release. Active secretion pathways can be targeted using endosomal inhibitors. Chloroquine, a well‐tolerated lysosomotropic agent, disrupts endosomal trafficking and subsequent DAMP export, positioning it as a potential therapy following traumatic injury or during early‐stage sepsis [240]. Passive release of DAMPs during regulated cell death can be attenuated by preserving membrane integrity. Ferrostatin‐1 exerts protective effects by inhibiting lipid peroxidation‐induced lytic death, acting upstream of the NINJ1‐mediated membrane rupture pathway to diminish the release of DAMPs and thereby alleviate acute lung injury [241].

Once liberated, circulating danger signals must be rapidly depleted to curtail their pro‐inflammatory effects. Host‐directed clearance utilizing Opsonic peptide 18, a multi‐DAMP scavenger, facilitates the phagocytic engulfment of diverse DAMPs, protecting against ischemia‐reperfusion injury by alleviating inflammation and tissue damage [242]. Comprehensive clearance strategies targeting both PAMPs and DAMPs simultaneously have also been developed. Extracorporeal blood purification techniques aim to remove cytokines, PAMPs, and DAMPs in sepsis [243]. However, basic extracorporeal blood purification lacks the selective clearance of PAMPs and DAMPs. To address this limitation, multitarget strategies such as telodendritic polymer‐based nanotraps (TD‐NTs) have been developed. It utilizes multivalent, charge‐based interactions to selectively capture inflammatory molecules with high efficiency (92%–99%) [244]. Notably, the efficacy of TD‐NT therapy is highly time‐dependent: early depletion may impair essential innate defenses, whereas delayed administration (3–8 h post‐insult) significantly improves survival, especially when combined with antibiotics [244]. Furthermore, RNases and nucleic acid‐binding microfibers enable specialized depletion of DNA‐ and RNA‐containing signals to prevent PRR hyperactivation [245].

Beyond clearance, direct neutralization of bioactive PAMPs and DAMPs has demonstrated therapeutic utility. Neutralization strategies utilize decoys or antibodies to “blind” circulating signals. Pathogen‐specific neutralization is exemplified by TCP‐25, a thrombin‐derived antimicrobial peptide. By sequestering LPS, TCP‐25 prevents CD14 interaction and TLR dimerization, effectively quenching downstream immune activation when delivered via functionalized hydrogels [246]. In bacterial sepsis, monoclonal antibodies targeting flagellin subtypes can restrict systemic dissemination and morbidity [247]. On the host side, S100A4‐neutralizing antibodies (e.g., 6B12) show potency in attenuating fibrosis [248]. However, the potential benefits of broad‐spectrum DAMP inhibition must be carefully calibrated against the risk of excessive immunosuppression. For example, combined blockade of DAMPs like MRP14 and HMGB1 may prove counterproductive [249]. Therefore, host‐directed therapy targeting only one type of DAMP can be sufficient to improve clinical outcomes in specific infectious contexts [249].

The final tier of intervention targets the interface between danger signals and their sensors. Small‐molecule inhibitors like Tasquinimod impede the binding of S100A8/A9 to TLR4 and RAGE, thereby reversing fibrotic phenotypes [138]. To preserve essential host defense, the tetramer P5779 specifically antagonizes the MD‐2/HMGB1 interaction while sparing LPS‐induced signaling, offering protection against sterile injury without compromising pathogen sensing [250]. Direct receptor antagonism, such as the TLR4 antagonist TAK‐242, remains a robust focus for acute inflammatory management [251]. Furthermore, in acute liver injury, blocking calcium release‐activated calcium channels prevents PAMP‐triggered vascular leakage and pulmonary edema by stabilizing the endothelial barrier [252].

#### 5.2.2 Harnessing DAMPs/PAMPs for Therapeutic Benefit

While the predominant therapeutic focus remains on inhibiting PAMP/DAMP‐mediated inflammation, the strategic activation of these pathways offers a powerful approach to overcoming immune tolerance in chronic infections and oncology. This context‐dependent duality allows clinicians to harness innate immune sensing to eliminate persistent pathogen infection or convert immunologically cold tumors into responsive lesions by repurposing ancient danger signatures as potent therapeutic stimuli. These strategies can be broadly categorized based on their primary targets: those enhancing innate immunity (boosting antimicrobial responses and inducing trained immunity) and those potentiating adaptive immunity (augmenting T cell‐mediated responses).

Strategies that harness PAMPs and DAMPs help restore host defense during infection. While the early host response to sepsis is characterized by hyperinflammation, this state is often accompanied by a concurrent failure to eradicate the primary pathogen and a heightened susceptibility to secondary infections [10]. To counteract this immunosuppression, an innovative vaccine technology, ciVAX, has been developed. ciVAX integrates PAMPs from inactivated pathogens, which are captured by an engineered opsonin, into a biomaterial scaffold with GM‐CSF and CpG. This assembly recruits and activates dendritic cells to prevent septic shock and enhance pathogen clearance [253]. In chronic viral management, such as for HBV, synthetic PAMP mimics are utilized to unmask viruses that typically evade host surveillance by maintaining cccDNA “invisibility”. For instance, PAMP mimics (e.g., 5‐triphosphate‐poly‐U/UC RNA) bridge this recognition gap, facilitating an IRF3‐dependent antiviral state that accelerates the decay of viral reservoirs when combined with standard nucleoside analogs [254]. Furthermore, the induction of trained immunity via fungal PAMPs, like chitin, has shown promise. Research found that chitin, a fungal PAMP, promotes the production of TNF‐α and IL‐6 through phagosome acidification, effectively “reprogramming” monocyte‐derived macrophages to enhance their long‐term antibacterial vigilance [255].

In the oncology landscape, the immunostimulatory properties of PAMPs and DAMPs have been extensively exploited, operating through effects on both adaptive immunity and innate immune training. The essential role of PAMPs in adaptive immunity is underscored by observations that cancer vaccination fails to elicit responses in the absence of gut microbiota, implicating microbial PAMPs as requisite co‐stimuli. Specific PRR agonists demonstrate remarkable synergy in the maturation of dendritic cells and in enhancing antigen‐presenting capacity. CpG oligonucleotides (TLR9 ligands) and poly(I:C) (TLR3 ligands) represent well‐characterized examples [256]. This dual‐stimulation approach significantly bolsters CD8^+^ T cell antitumor efficacy, particularly during periods of metabolic stasis. Bio‐inspired delivery systems, such as bacteria‐derived outer membrane vesicles, exploit the natural PAMP‐rich architecture of microbes to precisely target tumor tissues [257]. These polysaccharide‐ and nucleic acid‐based adjuvants provide the necessary “second signal” for T cell activation while driving metabolic reprogramming of immune cells, ensuring sustained therapeutic responses in vaccine design and glioma models.

Deliberate ICD induction converts the tumor microenvironment from immunosuppressive to immunostimulatory by promoting DAMP release. Gas plasma and gaseous signaling molecules, notably nitric oxide, trigger potent ICD effects through endoplasmic reticulum stress and mitochondrial dysfunction, promoting emission of DAMPs that serve as danger signals and elicit robust immunological protection against tumor rechallenge [258, 259]. Spermidine similarly facilitates DAMP liberation, alleviating immune surveillance and enhancing tumor recognition [260]. Furthermore, nanoscale metal‐organic frameworks (nMOFs) enable the synchronized, in situ delivery of PAMPs alongside liberated tumor antigens and DAMPs, thereby creating a personalized vaccine directly within the TME [261].

Beyond adaptive immunity, PAMPs and DAMPs exert therapeutic effects in oncology through innate immune training. Treatment with β‐glucan engages the Dectin‐1/mTOR/HIF‐1α axis, inducing a metabolic shift that polarizes macrophages toward a pro‐inflammatory M1‐like phenotype and enhances NK cell‐mediated cytotoxicity [262, 263]. This innate memory not only enhances phagocytic clearance of malignant cells but also facilitates the recruitment of mature DCs, thereby bridging the innate‐adaptive divide for comprehensive tumor surveillance [264].

## 6 Challenges, Limitations, and Future Perspectives

While the evolving understanding of PAMP and DAMP biology has provided profound insights into both immune regulation and the modulation of nonimmune cellular dynamics, several critical challenges and limitations remain unresolved.

First, conventional models often oversimplify the spatiotemporal complexity of danger signaling in vivo. A central theme of this review is the contextual duality of danger signals: although PAMPs and DAMPs evolved to protect the host by driving pathogen clearance and tissue repair, their dysregulation can fuel chronic inflammation and tissue damage. This duality is especially evident during severe infections, where the very immune responses necessary for pathogen elimination can culminate in systemic immunopathology and organ dysfunction. Similarly, in the tumor microenvironment, these signals act as a double‐edged sword. They can either prime robust antitumor immunity or foster an inflammatory niche that promotes tumor progression and immune evasion. While recent studies indicate that this context‐dependency is governed by molecular properties (such as redox states and other posttranslational modifications), signal concentration, exposure duration, and the local tissue microenvironment, a comprehensive understanding of this duality remains elusive. Furthermore, although the synergistic and antagonistic interactions between PAMPs and DAMPs have been outlined here, the precise molecular mechanisms underpinning their crosstalk require deeper investigation. It remains largely unclear how these signals integrate to cooperate or compete within distinct cellular landscapes.

Second, from a diagnostic perspective, the clinical utility of these danger signals is constrained by substantial methodological limitations. Standard detection platforms primarily quantify total protein pools and remain largely blind to the functionally distinct isoforms that govern specific biological activities. Furthermore, the rapid extracellular degradation of these molecules and their subsequent dilution in the peripheral circulation significantly diminish their accuracy and reliability as real‐time proxies for localized tissue damage.

Third, therapeutic targeting of the PAMP/DAMP axis necessitates a delicate clinical balance. In hyperinflammatory states, such as sepsis or acute autoimmune disorders, the broad‐spectrum neutralization or aggressive clearance of danger signals may inadvertently compromise the host's fundamental ability to combat primary or secondary infections. Conversely, in the context of cancer immunotherapy, the deliberate induction of immunogenic cell death or trained immunity must be carefully titrated to prevent excessive systemic inflammation or drug‐induced autoimmunity. Consequently, the extent of signal clearance, the precise therapeutic window, and the underlying disease context are critical variables that must be rigorously addressed during drug development. Future therapeutic strategies must evolve from broad inhibition toward precise, time‐sensitive modulation of these pathways.

Moving forward, overcoming these translational barriers requires a fundamental shift toward high‐resolution, multidimensional methodologies. First, the integration of single‐cell multi‐omics (encompassing transcriptomics, epigenomics, and metabolomics) will enable the identification of rare sentinel cell subpopulations that orchestrate PAMP/DAMP responses, potentially uncovering novel therapeutic checkpoints. Second, spatial transcriptomics and multiplexed imaging can map the in situ distribution of danger signals and their cognate receptor activation within tissues, providing a detailed view of regional heterogeneity that remains fundamentally unattainable through conventional bulk tissue analysis. On the therapeutic front, advancing precision nanomedicine and targeted delivery systems will facilitate the spatiotemporally controlled release or sequestration of these molecules. Such innovations will ultimately maximize therapeutic efficacy while safeguarding systemic immunological homeostasis.

## 7 Conclusion

The past three decades have seen significant progress in understanding how the immune system distinguishes between safety and danger. From Janeway's initial concept of pathogen‐associated molecular patterns to Matzinger's danger theory and the subsequent discovery of alarmins, this field now integrates immunology, cell biology, and clinical medicine. This review synthesizes these concepts to demonstrate that PAMPs and DAMPs operate not as isolated triggers, but as interconnected components of a systemic signaling network. While this network is necessary for immune responses, tissue homeostasis, and interorgan communication, its dysregulation drives various diseases. Future research should focus on translating these mechanistic insights into targeted therapies. Transitioning from broad‐spectrum interventions to precise, context‐dependent modulation will be necessary to resolve pathological inflammation or overcome immune tolerance without impairing the host's basic defenses.

## Author Contributions

**Xuanxuan Yu**: conceptualization, writing – original draft, writing – review and editing. **Yuqin Jin**: writing – original draft, writing – review and editing. **Baochao Li**: writing – review and editing. **Jie Deng**: writing – review and editing, funding acquisition. **Yiwen Zhou**: writing – review and editing, supervision. **Jinglun Zhang**: writing – review and editing, funding acquisition. **Huang Li**: conceptualization, writing – review and editing, supervision, funding acquisition.

All authors have given approval to the final version of the manuscript.

## Funding

This work is financially supported by the Key Project of the Health Commission of Jiangsu Province (ZD2022025); “2015” Cultivation Program for Reserve Talents for Academic Leaders of Nanjing Stomatological School, Medical School of Nanjing University (0223A101); National Natural Science Foundation of China (82201063; 82401077); Natural Science Foundation of Jiangsu Province (BK20240266).

## Ethics Statement

The authors have nothing to report.

## Conflicts of Interest

The authors declare no conflicts of interest.

## Data Availability

The authors have nothing to report.
